# Occurrence and sequence of Sphaeroides Heme Protein and Diheme Cytochrome C in purple photosynthetic bacteria in the family *Rhodobacteraceae*

**DOI:** 10.1186/1471-2091-11-24

**Published:** 2010-06-29

**Authors:** Terry E Meyer, John A Kyndt, Michael A Cusanovich

**Affiliations:** 1 Department of Chemistry and Biochemistry, University of Arizona, Tucson Arizona 85721 USA

## Abstract

**Background:**

Sphaeroides Heme Protein (SHP) was discovered in the purple photosynthetic bacterium, *Rhodobacter sphaeroides*, and is the only known c-type heme protein that binds oxygen. Although initially not believed to be widespread among the photosynthetic bacteria, the gene has now been found in more than 40 species of proteobacteria and generally appears to be regulated. *Rb. sphaeroides *is exceptional in not having regulatory genes associated with the operon. We have thus analyzed additional purple bacteria for the SHP gene and examined the genetic context to obtain new insights into the operon, its distribution, and possible function.

**Results:**

We found SHP in 9 out of 10 strains of *Rb. sphaeroides *and in 5 out of 10 purple photosynthetic bacterial species in the family *Rhodobacteraceae*. We found a remarkable similarity within the family including the lack of regulatory genes. Within the proteobacteria as a whole, SHP is part of a 3-6 gene operon that includes a membrane-spanning diheme cytochrome b and one or two diheme cytochromes c. Other genes in the operon include one of three distinct sensor kinase - response regulators, depending on species, that are likely to regulate SHP.

**Conclusions:**

SHP is not as rare as generally believed and has a role to play in the photosynthetic bacteria. Furthermore, the two companion cytochromes along with SHP are likely to function as an electron transfer pathway that results in the reduction of SHP by quinol and formation of the oxygen complex, which may function as an oxygenase. The three distinct sensors suggest at least as many separate functional roles for SHP. Two of the sensors are not well characterized, but the third is homologous to the QseC quorum sensor, which is present in a number of pathogens and typically appears to regulate genes involved in virulence.

## Background

SHP was discovered more than 24 years ago in the purple photosynthetic bacterium *Rhodobacter sphaeroides *[1]. Based on the SHP amino acid sequence [2] and three-dimensional structure [3], it is a member of the cytochrome c_4 _subfamily of class I cytochromes, which are characterized by a 5-10 residue insertion in front of the heme binding site and/or the occurrence of the sixth heme ligand within a helix. SHP is usually high-spin in all but four species and, in the oxidized form, it has a sixth ligand which dissociates upon reduction. It should be noted that SHP is the only heme protein that has an asparagine sixth ligand, which is the case for 33 out of 42 named species and environmental samples, of the remainder, five have aspartic acid, and four histidine (that also dissociates upon reduction [4]). All but four homologs of SHP have a cystine disulfide immediately after the sixth ligand, and when the *Methylophilus methylotrophus *cytochrome c" (an SHP homolog) disulfide is reduced and alkylated, the sixth ligand no longer dissociates upon heme reduction [4] suggesting that the disulfide introduces an element of rigidity. The unusual switching of ligands in SHP additionally has been observed in bacterial cytochrome c peroxidase (BCCP) [5], which is also related to cytochrome c_4_.

A unique property of SHP is that the reduced protein forms a relatively stable oxygen complex [1,2,6], something that had previously been observed only in the globins and in the cytochromes P450, and is now known to occur in bacterial oxygen sensors as well, including the BjFixL sensor kinase [7] and the EcDosH diguanylate cyclase [8]. In addition to the SHP from *Rb. sphaeroides*, the homolog from *Allochromatium vinosum *also binds oxygen [9], although that from *Mp. methylotrophus*, which has a histidine sixth ligand like hemoglobin, does not [4]. Reduced SHP binds the usual exogenous heme ligands, carbon monoxide, nitric oxide, azide, and cyanide [1,2]. Furthermore, reduced SHP appears to bind nitrogenous bases such as hydroxylamine, bis-tris-propane, HEPES, Tris, and taurine [1,2]. These properties suggest that the function of SHP is related to ligand binding and that it may have a role as an oxygenase. Recently, *Rb. sphaeroides *SHP has been reported to have nitric oxide dioxygenase activity [10], which is consistent with this hypothesis.

The gene sequence of *Rb. sphaeroides *SHP [11] indicates that it is located in a three-gene operon including a soluble diheme cytochrome c (sDHC) and a membrane-spanning cytochrome b (CytB)/membrane diheme cytochrome c (mDHC) chimera, which are likely to form an electron transfer pathway from a quinol via CytB and the two DHCs to SHP. The complete genome sequences of *Rb. sphaeroides *strains 2.4.3, 2.4.1, 2.4.9, and KD131 [12,13] indicate that SHP and the operon are present only in the last three. However, the 16S rRNA from the SHP negative strain, 2.4.3, contains 20 substitutions compared to the type strain 2.4.1 and differs by only two bases from what is currently recognized as a separate species called *Rhodobacter azotoformans *suggesting that strain 2.4.3 is not a *Rb. sphaeroides*. It should be noted that approximately 3% (or 45 substitutions) in any pair of rRNA are at the borderline for acceptance of separate species [14].

**Figure 1:**
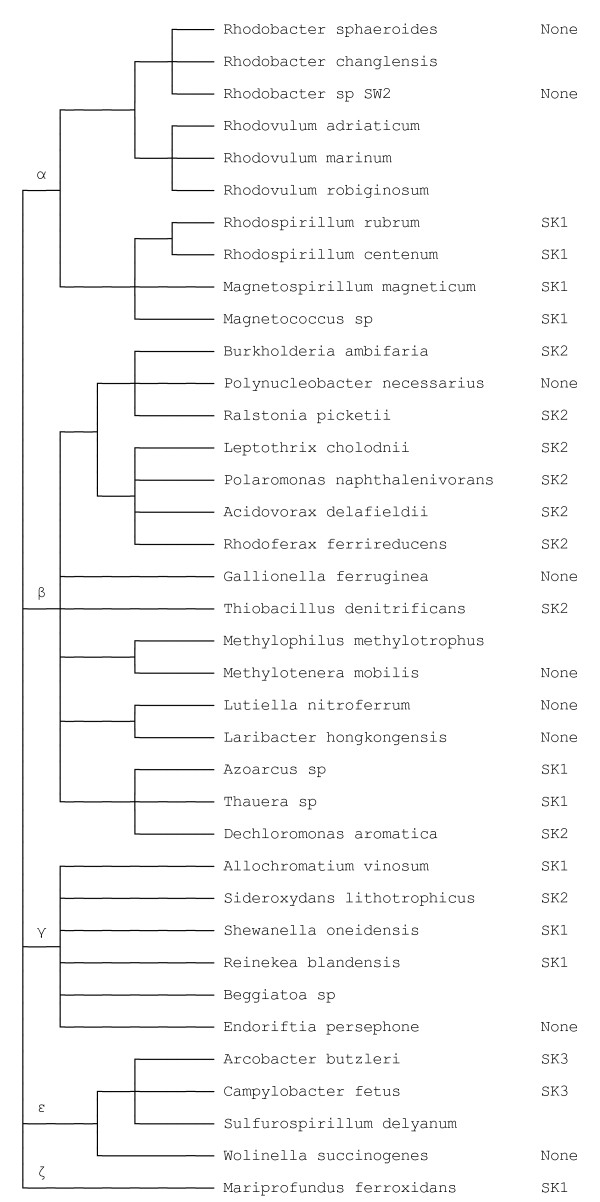
**Relationship among proteobacterial species that have the SHP operon**. The figure is based upon Bergey's Manual of Systematic Bacteriology, which is essentially a 16S rRNA based classification [23]. The five classes are indicated by greek letters. Species that have SK1, SK2, or SK3 regulatory genes as part of the SHP operon are indicated. There are genome sequences for all but *Mp. methylotrophus, Beggiatoa sp*., and the *Rhodobacteraceae *in this study for which there is no information on regulation. Additional closely related SHP-containing species, *Bh. multivorans, Bh. vietnamensis, Bh. phymatum, Bh. glumae*, *Sh. putrefaciens, Sh. baltica*, *Ms. magnetotacticum, Ms. gryphiswaldense*, and *Tc. tepidum*, are not shown because of space limitations.

At least 41 named species of proteobacteria in addition to *Rb. sphaeroides *are now known to contain SHP genes including the purple photosynthetic bacteria *Rhodospirillum rubrum*, *Rhodospirillum centenum, Allochromatium vinosum*, and *Thermochromatium tepidum*. Figure 1 shows the relationships among the species known to have SHP genes based on Bergey's Manual. Most species contain the three gene cluster, however the CytB is usually not found as a chimera with mDHC as was found with *Rb. sphaeroides*, with the exceptions noted below. In fact, the majority of species, except for *Rb. sphaeroides *and a few others, contain a pair of sensor kinase - response regulatory (SK/RR) genes as part of the gene cluster. These sensor kinases display one of three different sensors depending on species (SKI, SK2, and SK3, see below and Figure 1), suggesting that there are at least as many functions for SHP.

In addition to *Rb. sphaeroides*, five other species contain a CytB/mDHC chimera, *Rhodoferax ferrireducens*, *Campylobacter fetus*, *Arcobacter butzleri*, *Sulfurospirillum deleyanum*, and *Wolinella succinogenes*, which are on three separate branches of the evolutionary tree (Figure 1). Moreover, sDHC is found as a stand-alone gene in some Desulfovibrios and cyanobacteria. On the other hand, *Methylophilus methylotrophus*, which has a very divergent SHP homolog, does not appear to have a DHC and the genome sequence of the related *Methylotenera mobilis *indicates that DHC is absent in this family. *Rhodospirillum rubrum *and *Magnetospirillum magnetotacticum *have a CytB/SHP chimera, but the DHC is a separate gene.

SHP is not an abundant protein in *Rb. sphaeroides *(expressed at only 10-15% of the cytochrome c_2 _and c' under photosynthetic conditions) and is known to be regulated in part by the two component sensor kinase/response regulator pair, PrrBA, presumably under microaerophilic to anaerobic photosynthetic conditions [15]. In addition to SHP, PrrBA regulates approximately 25% of the genes in *Rb. sphaeroides*. However, prrBA is located elsewhere on the chromosome and there are no regulatory genes located within the SHP operon, unlike the majority of species studied to date (Figure 1). This raises the question whether SHP may have a different function in *Rb sphaeroides*. It is also possible that it has no function, or may have been acquired by gene transfer, perhaps from one of the species that have the unusual CytB/mDHC chimera similar to that of *Rb. sphaeroides*.

Given the wide distribution of SHP, its unique oxygen binding properties, apparent electron transfer arrangement (Cytb to SHP via DHC) and possible oxygenase function, it is of interest to better understand why SHP is not found in more photosynthetic species, and why *Rb. sphaeroides *is one of the few that do not contain regulatory genes as part of the operon. To this end, we prepared PCR probes for SHP, sDHC, and mDHC and were successful in isolating SHP genes from eleven out of twenty strains of photosynthetic bacteria examined in the family *Rhodobacteraceae*. Surprisingly, we did not find regulatory genes at all. Analysis of the gene context of SHP indicates at least three separate functional roles for SHP are maintained throughout the protobacteria.

## Methods

### Bacterial cultures

*Rhodobacter sphaeroides *strains TJ4, JB15, FY, SCJ, IL106, and 2.4.18 were from our collection. The first two were isolated by Dr. Paul Weaver and Dr. Robert Bartsch. *Rhodobacter *sp. strain TJ12 is another Weaver isolate that has not yet been completely characterized microbiologically, but is likely to be a new species based upon a 36-base difference in its 16S rRNA from *Rhodobacter capsulatus*. *Rb capsulatus *strains 2.3.1 and SP108 as well as *Rhodovulum sulfidophilum *strains W4 and BSW8, were also from our collection. *Rhodobacter blasticum *DSM2131, *Rhodobacter veldkampii *DSM11550, *Rhodovulum adriaticum *DSM2871, *Rhodovulum iodosum *DSM12328, *Rhodovulum euryhalinum *DSM4868, and *Rhodovulum imhoffii *DSM18064 were obtained from the German Collection of Microorganisms and Cell Cultures (DSMZ), Braunschweig. *Rhodobacter *sp. SW2 was a gift from Drs. Diane Newman and Alexandre Poulain, California Institute of Technology. *Rhodobacter changlensis *JA139 and *Rhodobacter vinaykumarii *JA123 were a gift from Dr. C. Ramana, Hyderabad India.

### Cytochrome PCR

PCR was performed using Taq polymerase and 40-45°C annealing temperature, only occasionally lowered to 30-35°C where no product was obtained at the higher temperature. Elongation was performed at 72°C for 1 min/kb. The SHP and DHC proteins are not highly conserved, it was thus a challenge to find regions of the sequence that could be used to design heterologous probes. We reasoned that the heme binding sites provided the best opportunity for cloning. The forward primer sDHC1F and the reverse primer sDHC2R based on the first and second hemes (**Additional file **1: **Table S1, and Additional file **2: **Figure S1**) were used to obtain most of the sDHC sequence from strains TJ4, JB15, SCJ, FY, and RI. They were also successful for mDHC from strains 2.4.18, IL106, JA139, and RE. We used the same forward primer sDHC1F but with the reverse primer mDHC2R to obtain most of the mDHC from strain FY. The forward primer SHPF, based on the single heme of SHP, was successfully used with the reverse primer sDHC2R to obtain an SHP/sDHC fragment for strains TJ4, JB15, FY, SCJ, RE, SW2, JA139, and DSM2871. Forward primer sDHC1F was successfully paired with reverse primer SHPR to obtain an mDHC/SHP product from strains TJ4 and SCJ. The specific forward primer mDHCF was paired with the specific reverse primer sDHCR to obtain an mDHC/SHP/sDHC product from strains TJ4, JB15, and SCJ. Primer mDHCF was also used with primer SHPR to obtain an mDHC/SHP product for strains FY, R18, and IL106. Forward primer CytBF was paired with reverse primer mDHCR to obtain a CytB/mDHC product from strains TJ4, JB15, SCJ, FY, 2.4.18, IL106, and JA139. The specific primers REmDHCF and REsDHCR were used to obtain an mDHC/SHP/sDHC product from strain RE. Likewise, the primers RCmDHCF and RCsDHCR were paired to obtain an mDHC/SHP/sDHC product from strain JA139. Primer SHPF was paired with sDHCRB to obtain an SHP/sDHC product for strains 2.4.18 and IL106.

### 16S rRNA PCR

The 16S rRNA sequences of the strains used in this study were determined as a control. Primer rRNA5PF was paired with primer rRNA3PR (**Additional file **1: **Table S1**) to obtain a 1422 base fragment. Because sequencing reactions return only 600 to 800 bases, primers from the center of the RNA, rRNAMF and rRNAMR, were designed for sequencing only.

## Results

### *Rb. sphaeroides* strains

**Figure 2:**
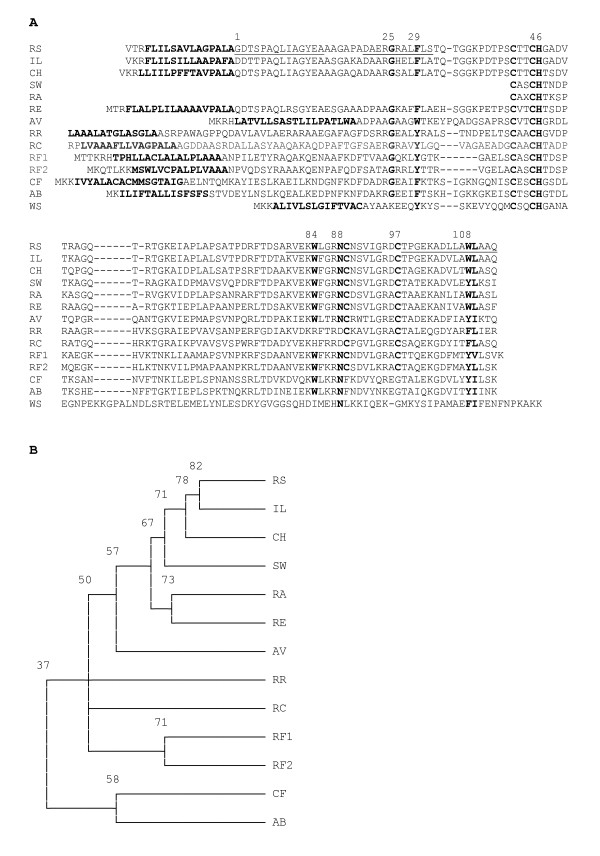
**A) Amino acid sequence alignment of SHP and B) Tree showing relationships among selected species (percentage identities for the branches are indicated)**. A) Symbols are: *Rhodobacter sphaeroides *strain 2.4.1 (RS), *Rb. sphaeroides *strain IL106 (IL), *Rhodobacter changlensis *(CH), *Rhodobacter *sp. strain SW2 (SW), *Rhodovulum adriaticum *(RA), *Rhodovulum marinum *strain RE (RE), *Allochromatium vinosum *(AV), *Rhodospirillum rubrum *(RR), *Rhodospirillum centenum *(RC), *Rhodoferax ferrireducens *(RF1 & RF2), *Campylobacter fetus *(CF), *Arcobacter butzleri *(AB), and *Wolinella succinogenes *(WS). The signal peptides as well as the amino acids mentioned in the text are in bold. Helices from the three-dimensional structure are underlined and important residues numbered according to *Rb. sphaeroides*.

**Figure 3:**
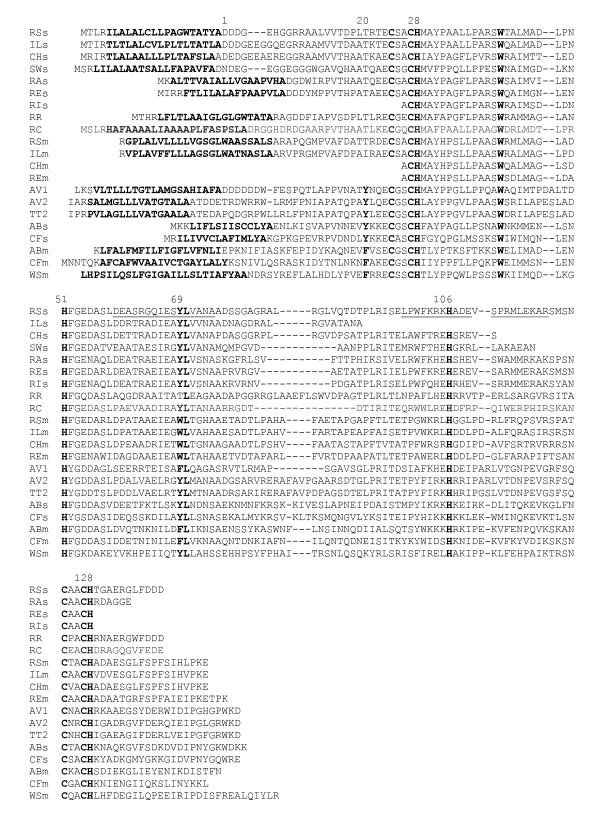
**Amino acid sequence alignment of DHC**. The same species and abbreviations as in Figure 1 followed by s for sDHC and m for mDHC or numbers 1 or 2 where there is more than one sDHC. Species not represented in Figure 1 are *Rhodovulum robiginosum *strain RI (RIs) and *Thermochromatium tepidum *(TT) for which we found sDHC but not SHP. Helices from the three-dimensional structure are underlined and important sequence positions numbered according to *Rb. sphaeroides*.

**Figure 4:**
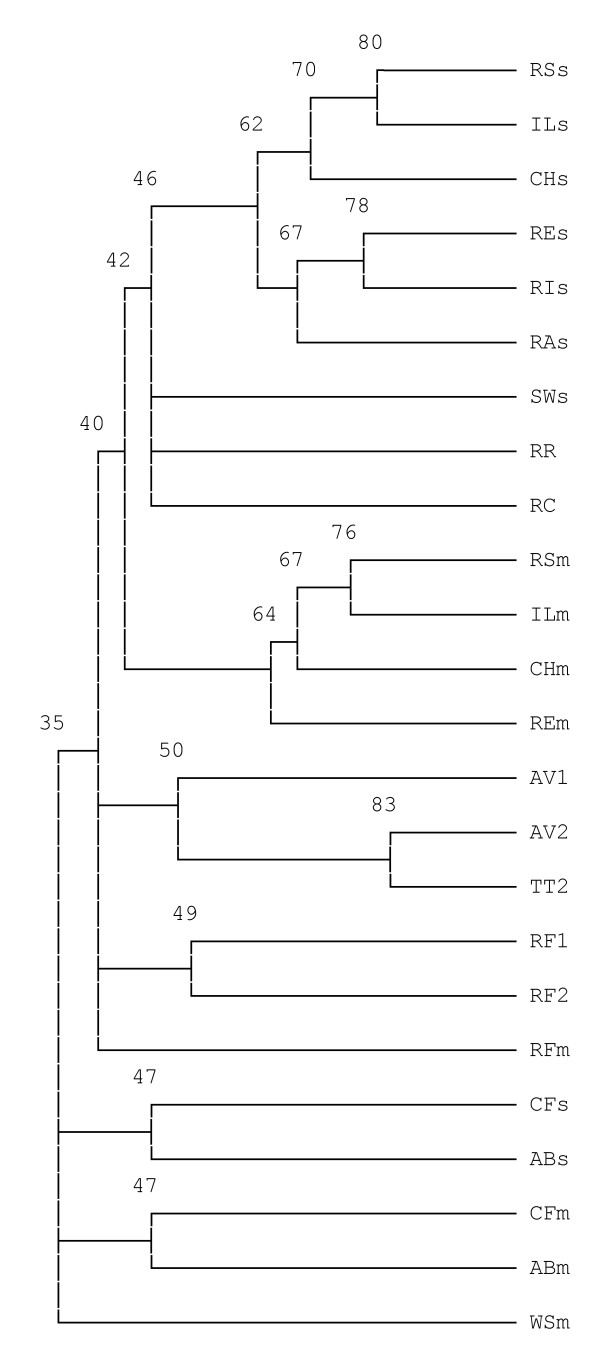
**A tree showing relationships among selected species of DHC (percentage identities for the branches are indicated)**. The same species and abbreviations as in Figure 3.

The first issue we addressed was whether SHP was present in just one or a few strains of *Rb. sphaeroides *or was characteristic of the species as a whole. Six new strains of *Rb. sphaeroides *were compared to four strains for which data currently exist (Table 1). Strains TJ4 and JB15 were isolated from Tijuana Estuary, San Diego County, California and the beach at La Jolla, California, respectively (about 20 miles distant). We found that their 16S rRNA sequences are identical to those of strains 2.4.1, 2.4.9, and KD131 (Table 1). We obtained PCR products for all three genes, CytB/mDHC, sDHC, and SHP, but could detect no differences in the 2 kb operon between strains TJ4 and JB15. However, strains 2.4.1, 2.4.9 and TJ4/JB15 differ from one another in the whole SHP operon by an average of 30 bases, whereas strain KD131 differs by 56 bases and has a 3 base insertion. Strain FY was isolated in France and has only an A565T substitution in its rRNA compared to strain 2.4.1. The strain FY SHP operon was found to have 41 differences and has a 15 base insertion in the signal peptide of the SHP gene identical to that of strain 2.4.9 although it does not have the 9 base insertion in sDHC which is also characteristic of strain 2.4.9. Strain SCJ was isolated from a sugar cane field in Jamaica and has only 2 base substitutions in its rRNA, A565T like strain FY and T579C. The SHP operon from strain SCJ differs by an average of 87 bases and it, along with strains TJ4, JB15 and KD131 contain a 3 base insertion near the start of the mature sDHC where strain 2.4.9 has a 9 base insertion. Strain 2.4.18 is from the Van Niel collection as were 2.4.1, 2.4.3, and 2.4.9 and there are just two base substitutions in the rRNA, A564G and A565T, the same as reported for strain IL106 which was isolated in Japan. We could detect only 5 differences in the SHP operon when comparing strains IL106 and 2.4.18. However, they are far more divergent in the SHP operon compared to the other strains we examined, containing 365 differences from strain 2.4.1, which is more than would be expected for a separate species. Strains IL106 and 2.4.18 also have the 9 base insertion in sDHC like strain 2.4.9. The amino acid sequences of SHP and the soluble and membrane bound DHC proteins of strain IL106 are shown in Figures 2, 3 and 4. Most of the base substitutions in the *Rb. sphaeroides *SHP operon occur in the third codon position and are therefore silent. However, the protein sequence of strain IL106 SHP is 82% identical to that of strain 2.4.1, the sDHC is 80% identical, the mDHC is 76% identical, and the CytB is 75% identical, consistent with the large number of base changes. The organization of the three genes and their spacing is similar to that of strain 2.4.1 (see Figure 4). We conclude from the above analysis that *Rb. sphaeroides *strains generally contain the SHP operon and that it was not recently acquired by gene transfer in just a few isolated strains. On the other hand, the large differences in the SHP operon of IL106 and 2.4.18 as opposed to the small number of substitutions in the rRNA compared with strain 2.4.1 or compared with the smaller numbers of changes in the strain SCJ operon, which has a similar variation in its rRNA, suggest more localized gene transfer within the genus or family. The sequences are available at [GenBank: FJ17738-FJ17746].

**Table 1:** *Rhodobacteraceae *species and strains

Name	B/mDHC	SHP	sDHC	16S rRNA
***Rb. sphaeroides *2.4.1**	+	+	+	0
***Rb. sphaeroides *2.4.9**	+	+	+	0
*Rb. sphaeroides *TJ4	+	+	+	0
*Rb. sphaeroides *JB15	+	+	+	0
***Rb. sphaeroides *KD131**	+	+	+	0
*Rb. sphaeroides *FY	+	+	+	A565T
*Rb. sphaeroides *SCJ	+	+	+	A565T, T579C
*Rb. sphaeroides *2.4.18	+	+	+	A565T, A564G
*Rb. sphaeroides *IL106	+	+	+	A565T, A564G
***Rb. sphaeroides *2.4.3**	-	-	-	20 bases
***Rb. capsulatus *SB1003**	-	-	-	58 bases
***Rhodobacter sp*. SW2**	+	+	+	60 bases
*Rb. changlensis *JA139	+	+	+	61 bases
*Rhodovulum marinum *RE	+	+	+	86 bases
*Rv. adriaticum *DSM2871	?	+	+	90 bases
*Rv. robiginosum *RI	?	?	+	103 bases

Strains for which the genome sequence has been determined are boldfaced. Species and strains examined in this study for the presence or absence of individual genes from the SHP operon (CytB/mDHC-SHP-sDHC) are indicated by a (+) or (-). A question mark indicates that they were not found with our probes, but could still be present. The numbers of base differences in the 16S rRNA of each strain and species is relative to *Rb. sphaeroides *2.4.1. Strains and species are arranged in order of increasing divergence.

### Other *Rhodobacteraceae *species

**Figure 5:**
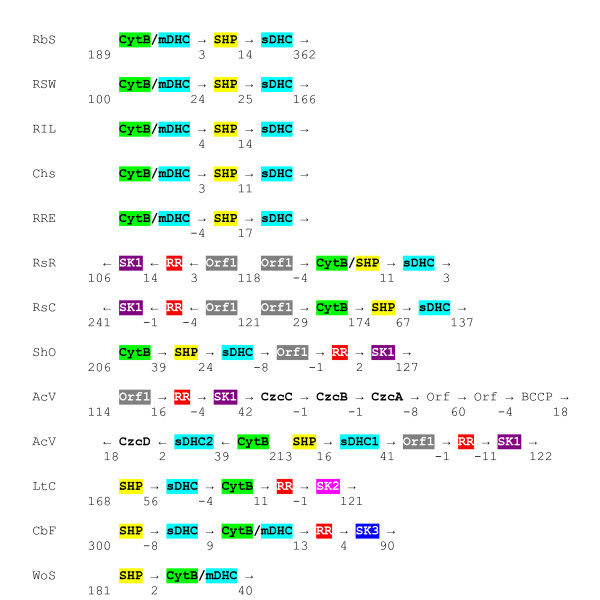
**Organization of SHP gene clusters**. RBS (*Rhodobacter sphaeroides *strains 2.4.1 and 2.4.9), RSW (*Rhodobacter *sp. SW2), RIL (*Rb. sphaeroides *strain IL106), Chs (*Rhodobacter changlensis*), RRE (*Rhodovulum marinum *strain RE), RsR (*Rhodospirillum rubrum*), RsC (*Rhodospirillum centenum*), ShO (*Shewanella oneidensis*), AcV (*Allochromatium vinosum*, a single cluster separated at the boundary between operons 1 and 2), LtC (*Leptothrix cholodnii*), CbF (*Campylobacter fetus*), and WoS (*Wolinella succinogenes*). CytB (Cytochrome b), mDHC (membrane diheme cytochrome c), SHP (sphaeroides heme protein), sDHC (soluble diheme cytochrome c), RR (response regulator), SK (sensor kinase), and Orf1 (periplasmic accessory protein) postulated to be part of the sensor, SK1. Slash indicates chimeric genes and presumably proteins as well. Spacing or degree of overlap between genes is indicated. Arrows show direction of transcription.

The second issue we addressed was whether the SHP operon was confined to *Rb. sphaeroides *or is more generally present in the photosynthetic *Rhodobacteraceae*. An sDHC protein was previously isolated and sequenced from *Rhodovulum adriaticum *[16], suggesting that the SHP operon might be present in species related to *Rb. sphaeroides*. We obtained a PCR product for *Rv. adriaticum *that includes both SHP and the sDHC, but have not yet been able to extend it to the CytB/mDHC (Table 1). A similar result was obtained with *Rhodobacter *strain SW2, which was isolated as an iron (II) oxidizing species [17]. *Rhodobacter changlensis *yielded nearly the entire operon from the beginning of CytB to the second heme of sDHC. *Rb. changlensis *was reported as a psychrophile growing from 5 to 32°C and is closely related to *Rhodobacter *strain SW2 and *Rb. sphaeroides *based upon fewer than 30 and 60 substitutions in its rRNA, respectively [18]. The *Rb. changlensis *SHP operon is organized very similarly to that of *Rb. sphaeroides *and includes the CytB/mDHC chimera as shown in Figure 5.

When we revived dried cultures (obtained from DSMZ) of *Rhodovulum euryhalinum *and *Rhodovulum iodosum*, the bacteria quickly grew, but the rRNA sequences indicate that they are not what was expected. In fact, the rRNA sequence from the "*Rv. euryhalinum *DSM4868" culture indicates that it differs by four bases from *Rhodovulum marinum *strain JA128. We will thus designate it *Rv. marinum *strain RE. The rRNA from the "*Rv. iodosum *DSM12328" culture differs by eight bases from that of *Rhodovulum robiginosum *strain DSM12329 which we designate strain RI. We were able to obtain only the sDHC gene from *Rv. robiginosum *strain RI. We had better results with *Rv. marinum *strain RE in that most of the SHP operon from the first heme of mDHC to the second heme of sDHC, including the SHP, was present. No regulatory genes were observed in any of the SHP operons of the *Rhodobacteraceae *(Figure 1).

PCR was negative for three strains of *Rhodobacter capsulatus*, SB1003, 2.3.1 and SP108*, Rhodobacter *TJ12, *Rhodobacter blasticum, Rhodobacter veldkampii, Rhodovulum imhoffii*, and two strains of *Rhodovulum sulfidophilum*, W4 and BSW8. As indicated by the less than complete recovery of the SHP operon from the species that gave a positive result, the negative results do not necessarily indicate absence of the genes except possibly in *Rb. capsulatus *strain SB1003, for which there is a genome sequence. It merely indicates that if the operon is present, it is too divergent to be identified by our probes. We conclude from these results that the SHP operon is present in at least half the photosynthetic species of *Rhodobacteraceae*.

### Sequence Comparisons

Additional questions of interest are whether the photosynthetic species with the SHP operon are more closely related than to the other proteobacteria, whether they evolved in concert, and whether they may be more closely related to the species with the CytB/mDHC chimera. We also questioned whether amino acid residues near the SHP heme were conserved, which would suggest similar functional roles. Translated amino acid sequences for the *Rhodobacteraceae *SHP and DHC genes are shown in Figures 2, 3 and 4, along with trees which indicate that the sDHCs are closely related as are the mDHCs, but the cross comparison indicates that they are as divergent from one another as are any of the 41 species previously characterized from proteobacteria (4 species have 2 SHPs and 3 species have 2 or 3 sDHCs). Additional species were not placed in the trees because the sequences were too divergent to give reliable results. The mean identity of all species of SHP is 45% with 6% standard deviation, indicating that only those data above 51% identity are highly significant. Those species below that cutoff are approaching a limit to change dictated by their function [19]. The mean of all species of DHC is 40% with 6% standard deviation, indicating that only data above 46% are significant. Most *Rb. sphaeroides *strains of SHP average 2 differences and the DHCs average 4. Based on the foregoing, we conclude the three genes of the operon evolved in concert within the *Rhodobacteraceae*. Moreover, there is no indication that they are more closely related to any of the non-photosynthetic species with the CytB/mDHC chimera.

Note that the signature residues for the N-terminal helix of SHP, G25 and Y29, are 8-13 residues in front of the heme binding site, which corresponds to a 5-10 residue insertion (a normal class 1 cytochrome has a 3 residue spacer). This is typical of the cytochrome c_4 _family of class I cytochromes. Likewise, the signature residues for the C-terminal helix, W108L109, follow the sixth ligand N89 by 19 residues (the normal class 1 signature has a 17 residue spacer). The 6 new SHPs reported here all have the C89-C97 disulfide assuming structural homology with *Rb. sphaeroides *[3]. All of the *Rhodobacteraceae *SHPs have a single residue deletion in the vicinity of residue 56, which is shared with *Thiobacillus denitrificans, Laribacter hongkongensis, Lutiella nitroferrum, Azoarcus*, and *Thauera*, suggesting that they may be more closely related than to the remaining species, although that is not obvious on a percentage basis. The purple photosynthetic bacterium from the family *Chromatiaceae, Allochromatium vinosum*, has a normal SHP, but those from the other purple photosynthetic bacterial family, the *Rhodospirillaceae, Rsp. rubrum *and *Rsp. centenum *are very different from those of the *Rhodobacteraceae *in that they have an Asp88 sixth ligand and substitution of Trp84 (Arg or His), which almost certainly will have functional consequences. We conclude from these two differences that there are likely to be at least two separate functional roles for SHP within photosynthetic species.

The sequences of the *Rhodobacteraceae *DHCs (Figure 3) show that the *Rhodobacter *and *Rhodovulum *species are distinct, which was also the case with the SHPs. The new sDHCs are also clearly delineated from the mDHCs on a percentage identity basis (Figure 4). In addition, the sDHC sequences are very different from those of species outside the family including those in which there has been gene duplication (for example, *Chromatium*). That is, the sDHC has reached a limit to change with respect to the mDHC, suggesting that they were duplicated before the *Rhodobacteraceae *began to evolve. Unlike SHP, there is no variation in the sixth heme ligands of the DHCs. The first heme domain of both mDHC and sDHC is a generic class I cytochrome not closely related to SHP or cytochrome c_4_. Thus, there is no insertion between the N-terminal helix and the heme and there are 17 residues between the sixth ligand, H51, and the signature residues Y69L70 of the C-terminal helix.

### Gene Organization

Another question to be addressed by comparative analysis was whether gene context could lead to inferences about regulation and functional role of SHP. However, gene context is not particularly significant unless it can be shown that a particular gene organization is shared by several species. The organization of the genes within the SHP operon is shown in Figure 5. CytB/mDHC is the first gene followed by SHP and ending with sDHC in all four *Rhodobacteraceae *species characterized here. The close association of these three genes, although not necessarily in the same order, is found in all but two of 41 species, indicating that they are functionally coupled and likely to form an electron transfer pathway. In *Rb. sphaeroides*, there are no other genes associated with the SHP operon.

In the two species of photosynthetic *Rhodospirillaceae*, *Rs. rubrum *and *Rs. centenum*, the gene order is the same as in *Rhodobacter*, but there is no mDHC, the SHP is fused to CytB in *Rs. rubrum*, and the first gene in the operon is an uncharacterized open reading frame (Orf1). In the opposite orientation, there is another operon starting with a homologous orf1 and including a response regulator and a sensor kinase (SK1). The presence of the highly homologous orfs on both sides of the promoter region indicates the possible presence of a divergent promoter and a functional coupling between the two operons. Interestingly, the two operons are combined in a single six-gene operon in non-photosynthetic species such as *Magnetococcus *sp., *Reinekea blandensis*, *Mariprofundus ferroxidans*, and *Shewanella oneidensis *(shown in Figure 5). This further indicates a functional coupling between the two operons. The SK1 sensor is always associated with Orf1, is found in many species, and is often part of the SHP gene cluster (in at least 15 of the 42 species). Because of the numbers of species involved, the association of regulatory genes with SHP is highly significant. Thus, it is reasonable to assume that the SHP genes are regulated in part by SK1. The presence of regulatory genes in association with the SHP operon in the *Rhodospirillaceae *but not the *Rhodobacteraceae *suggests a different function for SHP in the two families of photosynthetic bacteria, as was proposed above based upon sequence substitutions in the SHPs.

In the photosynthetic *Chromatiaceae*, represented by *Allochromatium vinosum*, there are at least 3 operons in a cluster including 17 genes. In the third operon, SHP and sDHC are associated with the same three regulatory genes as in the *Rhodospirillaceae *(Orf1/RR/SK1). In the second operon, CytB and another sDHC are situated in the opposite orientation and associated with a heavy metal transport gene (CzcD, which stands for Cadmium, Zinc, and Cobalt). In addition, preceding the two SHP related operons, is a nine gene operon starting with the same three regulatory genes as associated with SHP, three additional heavy metal transport genes (CzcABC), two open reading frames, and a peroxidase (BCCP). CzcD is also associated with the SHP operon in another member of the family, *Thermochromatium tepidum*, and at least two other species, *Lutiella nitroferrum *and *Thauera *sp. Therefore, it is possible that there is a functional connection between SHP, the heavy metal transport genes, and the regulatory genes. These findings are consistent with different functional roles for SHP in the three major families of purple photosynthetic bacteria *Chromatiaceae*, *Rhodobacteraceae *and *Rhodospirillaceae*.

## Discussion

Since its discovery, it has been established that the SHP gene occurs in over 42 species of divergent proteobacteria. Nevertheless, before initiation of this study, the protein had only been observed in one out of about 16 species of non-sulfur purple bacteria that had been examined, i.e. in *Rb. sphaeroides*, and thus appeared to be an anomaly. We have now shown that SHP is not confined to one or a few strains of *Rb. sphaeroides*, but is present in 9 out of 10 strains studied to date and the one negative strain, 2.4.3, is likely to be a separate species called *Rb. azotoformans*. Thus, the presence of SHP is not due to an isolated occurrence of gene transfer from some other species, but is truly characteristic of *Rb. sphaeroides*. If SHP had resulted from gene transfer, it may not have been functional or important to the survival of the species and absent from at least some strains. We now know that is not likely to be the case and the fact that the SHP operon is found in five related species of *Rhodobacteraceae *suggests that it is functional and of selective advantage to these species. Not only is the SHP operon present in other species of *Rhodobacteraceae*, but the relationships shown in Figures 2, 3 and 4 indicate that the SHPs and DHCs are more closely related to one another than to any other species, also indicating that there has been no recent gene transfer from outside the family. Our studies furthermore show that DHC was duplicated in the *Rhodobacteraceae *prior to speciation. It is impossible to say at this point how many times DHC was duplicated since there are species that have several copies of SHP and of sDHC. Gene duplication thus appears to be relatively common in the SHP operon.

We have shown that *Rb. sphaeroides, Rb. changlensis*, *Rv. marinum*, and presumably other *Rhodobacteraceae *(in the alpha class) have the CytB/mDHC chimera as part of the SHP operon. The only other species with a similar chimera are *Rf. ferrireducens *(in the beta class) and *Cb. fetus, Ab. butzleri*, and *Wo. succinogenes *(in the epsilon class). The latter three species are related, but all four have significant differences to one another and to *Rb. sphaeroides *such as the position of the SHP gene before the CytB (Figure 5). Furthermore, *Wo. succinogenes *does not have an sDHC, *Cb. fetus *and *Ab. butzleri *have regulatory genes as part of the operon, and none of these three SHPs have the C89-C97 disulfide. In terms of percentage identity, the *Campylobacter *and two related SHP operons are among the most divergent as shown in Figures 2 and 3. It is therefore likely that the chimera does not have a single origin.

Although evolution and gene transfer are important, we were particularly interested in what comparative analysis could tell us about the function of SHP in photosynthetic bacteria. One of the first clues to the function of SHP is the rare occurrence of ligand-switching upon reduction followed by oxygen binding. SHP is the only c-type heme protein known to bind oxygen [1,2,6,9]. Among the b-type heme proteins, only the globins and cytochrome P-450 plus a few heme-containing sensor kinases and diguanylate cyclases form stable oxygen complexes, and in these cases, it is functionally important. By analogy to P-450, SHP can be postulated to be an oxygenase, which hydroxylates an unknown substrate. SHP has in fact been reported to oxidize nitric oxide to nitrate [10] as has been observed with other oxygen-binding heme proteins [20]. Only *Ac. vinosum *[9] and *Rb. sphaeroides *[1] SHP have been tested and shown to bind oxygen, which is consistent with the lack of significant amino acid substitutions in the vicinity of the heme. On the other hand, *Rs. rubrum *and *Rs. centenum *SHPs have an N88D substitution of the sixth heme ligand and W84R and W84H substitutions that presumably stabilize Asp 88, respectively. N88 and W84 are strongly correlated in other species and are closely situated at the distal side of the heme. In computer models, the H84 substitution is close enough to bind to the heme iron, raising the interesting question whether it actually does so. In any case, the amino acid substitutions may be sufficient to preclude oxygen binding and suggest a different functional role for the *Rhodospirillaceae *SHPs.

The second clue to the function of SHP comes from the equally rare occurrence of binding organic amines, among which are Tris, HEPES, bis-tris-propane, hydroxylamine, and taurine [2]. It is unlikely that the heme iron simultaneously binds both oxygen and organic amines, but there could be a separate binding site for co-substrate near the heme-bound oxygen which is rather open and exposed to solvent [3].

The third clue to the function of SHP comes from the genetic context of the SHP gene. SHP is in fact part of a three-gene operon along with a CytB/mDHC chimera and an sDHC. That is not only true for *Rb. sphaeroides *and the *Rhodobacteraceae*, but is generally true for the majority of SHP species as shown in Figure 5. The membrane-spanning cytochrome b has binding sites for two hemes and is related to cytochromes b that interact with quinones such as the membrane subunit of formate dehydrogenase [21]. It is thus likely that a quinol reduces the CytB which reduces the mDHC and the sDHC, which then reduces SHP. The latter reaction has been clearly demonstrated for *Rb. sphaeroides *[6]. That the diheme cytochromes could provide both necessary electrons to SHP to activate oxygen, is consistent with SHP functioning as an oxygenase.

The fourth clue to the function of SHP also comes from the genetic context. The majority of SHP operons include sensor kinase and response regulatory (SK/RR) genes with the exception of the three *Rb. sphaeroides *strains and *Rhodobacter *SW2 for which there are whole genome sequences and a few others as shown in Figure 5. There are three types of sensors associated with the SHP operons (Figure 1), which we will describe below. Of these three, the first two (SK1 and SK2) are equally common (74 and 60 examples out of *ca *700 species known to date) and the third (SK3) rare (*Arcobacter butzleri *and *Campylobacter fetus*, Figure 1). The first two occur both within the SHP operon (15 and 12 examples) and in other contexts. Only SK1 is associated with SHP in the two photosynthetic families, *Rhodospirillaceae *and *Chromatiaceae*.

The first sensor kinase (SK1) and the accessory protein (orf1) are strongly correlated, both in the SHP operons and in other contexts in which they are found. *Vibrio *species are the only pathogens of which we are aware that have the SK1 sensor kinase. The SK1 sensor can be recognized by variations of the SGVYWQ and RSRSLWD motifs in the periplasmic domain. There are SK1 (RSP3512) and Orf1 (RSP3510) genes in *Rb. sphaeroides *strain 2.4.1 and they are located on chromosome II. On the other hand, SHP (RSP2021) is on chromosome I, but there is no known functional relationship. However, the presence of regulatory genes as part of the SHP operon in most species and the association of *Rhodospirillaceae *and *Chromatiaceae *SHPs with SK1 suggest that at least some of the *Rhodobacteraceae *SHPs may be regulated in part by SK1 as well. Since this study was initiated, the genome of *Rhodobacter *SW2 has been determined (Joint Genome Institute). These results show that while PrrBA genes are present, there are no regulatory genes associated with SHP, and SK1 and SK2 are absent.

The SK2 sensor is related to the enterohemorrhagic *E. coli *QseC quorum sensor that responds to autoinducer 3 (AI3, structure unknown) and also responds to epinephrine and norepinephrine from the host [22], both of which are organic amines. The QseC homologs include the important pathogens: *Actinobacillus*, *Bordetella, Escherichia, Haemophilus, Legionella, Pasteurella, Pseudomonas, Salmonella, Vibrio*, and *Yersinia*. Several of the pathogenic *Burkholderia *species (5 out of *ca *14) contain the SHP operon including the SK2 sensor kinase. Presumably, these pathogenic bacteria respond to the concentration of AI3 excreted into the environment or to epinephrine produced by the host to activate virulence genes. Either the SHP operon is one of several that may be regulated by quorum sensing or may be turned on specifically to inactivate the autoinducer produced by competitors. Although there is an SK2 gene in *Rb. sphaeroides*, it is located elsewhere on the chromosome (RSP3431) and there is no known functional connection with SHP, yet its involvement in regulation of the *Rb. sphaeroides *SHP operon cannot be excluded at this time.

The third type of sensor kinase, SK3, is unique to *Cb. fetus *and *Ab. butzleri *and not found in *Wo. succinogenes *or *Rb. sphaeroides *or any other species of which we are aware. The more divergent SHP operon organization and the presence of a different SK indicate that the function of SHP is likely to be different in this group compared to the previous two, which may be regulated in part by SK1 and/or SK2.

## Conclusions

SHP is not as unusual in purple photosynthetic bacteria as previously supposed since it is present in approximately half the species tested and was not acquired by recent gene transfer. The DHC was duplicated and fused to CytB within the *Rhodobacteraceae*, an event that has occurred more than once during evolution. Furthermore, the CytB and DHC along with SHP are likely to function as an electron transfer pathway that results in the reduction of SHP by quinol and formation of the oxygen complex. One postulated role is that SHP hydroxylates an unknown substrate by analogy with P450. That there could be a role for an oxygenase in *Rb. sphaeroides *is in itself surprising, but the photosynthetic lifestyle does not lend itself to complex organic transformations, which makes it even more unusual. There are three distinct sensor kinases associated with SHP in various species that suggests as many functional roles. One of the sensors is homologous to the QseC quorum sensor, which is present in a number of pathogens. It is clear that a better understanding of SHP, its regulation, and function could be important in controlling those pathogens.

## Authors' contributions

TEM and JAK designed and performed the experiments, TEM drafted the manuscript with the help of JAK and MAC. All authors contributed equally to the interpretation of the data. All authors read and approved the final manuscript.

## Supplementary Material

Additional file 1 **Table S1**. Oligonucleotide primers used to clone the genes of the SHP operon. Click here for file

Additional file 2 **Figure S1**. Location of the oligonucleotide primers and their orientation relative to the genes of the SHP operon. Click here for file
