# Supplementary material for: Occurrence and sequence of Sphaeroides Heme Protein and Diheme Cytochrome C in purple photosynthetic bacteria in the family Rhodobacteraceae
Source: BMC Biochem. 2010 Jun 29;11:24. doi: 10.1186/1471-2091-11-24 (PMC2909971; doi:10.1186/1471-2091-11-24)
Supplement: Additional file 1 — Table S1. Oligonucleotide primers used to clone the genes of the SHP operon. [file 1471-2091-11-24-S1.DOC]

**Additional file 1: TABLE S1**. Oligonucleotide primers used to clone the genes of the SHP operon. These are shown on the top line. The corresponding amino acid sequence is shown on the bottom line. The primers used to clone and sequence the 16S rRNA are also shown.

| sDHC1F | GC**S**TGCCA**Y**ATGGC**S**TA**Y**C  A C H M A Y P |
| --- | --- |
| sDHC2R | **R**TGGCA**S**GC**S**GCGCA**R**TT  H C A A C N |
| sDHCR | CGCGGCTCGCCTCGTCG  G R S A E D |
| mDHCF | CTGGAAGCGCCTGCACG  W K R L H |
| mDHC2R | GC**R**TG**R**CA**S**GC**S**GT**R**CA**S**G  A H C A T C T |
| mDHCR | C**K**CGCATCCTC**K**CCGAAATGGTC  R A D E G F H D |
| SHPF | CCTGC**R**CCA**S**CTGCCAC  C T T C H |
| SHPR | GTGGCAG**S**TGG**Y**GCAGG  H C T T C |
| CytBF | CC**S**ATCCG**S**CT**S**TT**Y**CACTGGTC  P I R L F H W S |
| REmDHCF | CCCCGGCCTGGGAACGGTTGCAC  P A W E R L H |
| REsDHCR | CATGTTCGCGCTTGAACCAGGGC  E H E R K F W P |
| RCmDHCF | CCCTTCTGGCGCAGCCGGCACGG  P F W R S R H G |
| RCsDHCR | GTTCGCGGGTGAACCATGCCAG  H E R T F W A L |
| sDHCRB | G**SKK**TGAACCA**N**GSCAG**Y**TC  R K F W P L E |
| rRNA5PF | GTTTGATCCTGGCTCAG |
| rRNA3PR | TACCTTGTTACGACTTCA |
| rRNAMF | CCAGTGGCGAAGGCGGC |
| rRNAMR | GGCATTCATCGTTTACG |
