# Supplementary material for: Occurrence and sequence of Sphaeroides Heme Protein and Diheme Cytochrome C in purple photosynthetic bacteria in the family Rhodobacteraceae
Source: BMC Biochem. 2010 Jun 29;11:24. doi: 10.1186/1471-2091-11-24 (PMC2909971; doi:10.1186/1471-2091-11-24)
Supplement: Additional file 2 — Figure S1. Location of the oligonucleotide primers and their orientation relative to the genes of the SHP operon. [file 1471-2091-11-24-S2.DOC]

Additional file 2: Figure S1. The three genes of the SHP operon and the primers used to clone them.

CytB mDHC SHP sDHC

___________________________________________________ _________________ _________________________

______ ______ ______ _______ _______ _______ ______ _______

CytBF mDHCR mDHCF mDHC2R SHPFR sDHC1F sDHCR sDHC2R
